# Associations between traumatic event experiences, psychiatric disorders, and suicidal behavior in the general population of Afghanistan: findings from Afghan National Mental Health Survey

**DOI:** 10.1186/s40621-022-00403-8

**Published:** 2022-10-06

**Authors:** Ajmal Sabawoon, Katherine M. Keyes, Elie Karam, Viviane Kovess-Masfety

**Affiliations:** 1 Department of Epidemiology, Mailman School of Public Health, Columbia University, New York, NY USA; 2 Institute for Development, Research, Advocacy & Applied Care (IDRAAC), Beirut, Lebanon; 3 Department of Psychiatry and Clinical Psychology, Faculty of Medicine, St. George Hospital University Medical Center University of Balamand, Beirut, Lebanon; 4 Université de Paris, LPPS, Boulogne-Billancourt, France; 5 Department of Psychiatry, McGill University, Montreal, Canada

**Keywords:** Suicide, Suicidal behavior psychiatric disorders, Trauma, Afghanistan, Gender

## Abstract

**Background:**

The role of traumatic event exposure and psychiatric disorders as central risk factors for suicidal behavior has been established, but there are limited data in high conflict regions with significant trauma exposures such as Afghanistan.

**Methods:**

A nationally representative, cross-sectional survey was conducted through systematic stratified random sampling in 8 regions of Afghanistan in 2017 (*N* = 4474). Well-validated instruments were used to establish trauma exposure, psychiatric disorders. Death preference, suicidal ideation, plan, and attempts were assessed.

**Results:**

In the total sample, 2.2% reported suicidal ideation in the past 12 months, and 7.1% of respondents reported that they had suicidal ideation at some point in their lives; 3.4% reported a suicide attempt. Women were at higher risk than men. All traumatic event exposures were strongly associated with suicidal behavior. Respondents who reported experiencing sexual violence were 4.4 times more likely to report lifetime suicide attempts (95% CI 2.3–8.4) and 5.8 times more likely to report past 12-month suicidal ideation (95% CI 2.7–12.4). Associations were strong and significant for all psychiatric disorders related to suicidal behavior. Respondents who met criteria for major depressive episodes (OR = 7.48; 95% CI 4.40–12.72), generalized anxiety disorder (OR = 6.61; 95% CI 3.54–12.33), and PTSD (OR = 7.26; 95% CI 4.21–12.51) had the highest risk of past 12-month suicidal ideation.

**Conclusion:**

Traumatic event exposures and psychiatric disorders increase risk of suicidal behavior in the Afghan general population; women are at high risk. Interventions to reduce trauma exposure, including expansion of a mental health workforce in the region, are critically important.

## Background

Suicidal behavior such as ideation about death and intentional suicidal self-harm are relatively common (Nock et al. 2008, 2010; Borges et al. 2010) and concerning for public health as such behavior may be indicative of underlying psychiatric disorders and distress, as well as a precursor to serious self-harm and death (Brown et al. 2000; Mann et al. 1999). Suicide death remains a major driver of mortality across the world, contributing to at least 700,000 deaths annually (World Health Organization 2021), of which an estimated 77% occur in low- and middle-income countries (World Health Organization 2021); suicidal ideation and behavior remain key risk factors for suicidal death (Cavanagh et al. 2003), and can be psychologically distressing even when not accompanied by a serious suicide attempt. Understanding drivers of suicidal behavior, especially in areas of the world that are understudied, therefore remains critical to global health and mental health.

Exposure to traumatic events is a strong and consistent risk factor for suicidal behavior (Beristianos et al. 2016; Lira et al. 2022; Wilcox et al. 2009). The severity of the resulting psychological distress after exposure to trauma varies as a function of the severity of the trauma exposure, social support available to the survivor, and pre-existing level of psychiatric burden or psychological distress (Asarnow et al. 2020; Krysinska and Lester 2010; Grandison et al. 2022). Yet there remain gaps in the literature on the prevalence, distribution, and psychiatric correlates of suicidal behavior and its association with trauma in low- and middle-income countries, especially in those with histories of war and conflicts as well as countries in which religious taboos may make mental health stigma and discussion of suicide, especially difficult. These gaps are important; we cannot assume that science of suicidal ideation and behavior from studies largely in high-income settings generalize across the world, especially to areas with different cultural and religious practices as well as unique trauma histories.

Suicide is highly discouraged as a matter of Islamic faith (Kazi and Naidoo 2016), and Islam may be the most influential among religions in reducing the number of suicides among its followers, with some rare exceptions (Chel’loob 2019). While religiosity is generally protective from fatal self-harm, low suicide rates may also reflect misclassification of suicide deaths as due to other causes given its stigmatized nature. Of special interest is a high rate of young females’ suicide and attempted suicide in Muslim-dominated Middle Eastern countries (Rezaeian 2010). This has been linked to the effect of traditional customs in the Middle East such as restrictive marriage customs and domestic violence, together with violent methods of suicide and elevated depressive rates among women. Furthermore, these countries have high rates of both internal and external migration due to poverty, unemployment, and armed conflict, which by itself may increase the rate of suicidal behavior. All these apply to the Afghan context, to different degrees along the multi-ethnic components of Afghanistan, and potential spatial correlation of geographic regions where they are living, intensity of terrorists’ attacks, and rural/urban situations.

Indeed, the population of Afghanistan has endured more than three decades of exposure to international and civil war and violence, resulting in ubiquitous exposure to often extreme levels of stress and bereavement, as well as strain due to destruction of economic, social, and cultural infrastructure (Alemi et al. 2014; Silove and Ventevogel 2022). Existing population-based research in Afghanistan is limited, but what has been done demonstrates a high burden of psychiatric disorders and traumatic event exposure (Kovess-Masfety et al. 2021a, b). Studies of suicidal behavior in Afghanistan are limited, in part perhaps due to the stigmatized nature of suicide. However, such information is critical to begin conducting surveillance on, both to inform the literature on suicidal behavior in a country with unique trauma exposures, as well as to build an evidence base for continued study. Indeed, Afghanistan is once again going through a period of change, with the withdrawal of US occupation from the region and reinstatement of Taliban government in 2021. Building a research infrastructure that attends to surveillance of mental health and suicidal behavior in the Afghan population will allow for assessment of burden and needs in the region and inform the broader literature about the effects of war and trauma within this understudied population.

The present study analyzes the largest dataset of psychiatric disorders, trauma, and suicidal behavior conducted to date in the Afghan general population to address three aims: first, we examine the burden of suicidal ideation and attempt, and demographic correlates including gender and age, in the Afghan general population; second, we examine the association with experiences of trauma and stressful experiences; third, we examine associations with psychiatric and substance disorders including mood and anxiety disorders, psychotic experiences, and tobacco and other drug use.

## Methods

### Sample

A cross-sectional household survey was implemented from April to October 2017 in each of the eight regions of Afghanistan: (1) Eastern; (2) South Eastern; (3) Southern; (4) Western; (5) North Western; (6) North Eastern; (7) Central Kabul; and (8) Central Bamiyan. A multi-stage stratified cluster sampling method was applied: in each region, two provinces were randomly selected totaling 16 provinces out of a possible 34. A random sampling of clusters within province was selected, based on maps of 320 clusters provided by the Central Statistical Organization. Within each cluster, 14 households were randomly selected and eligibility criteria assessed. In the household, a randomized adult selection was based on Kish selection before starting the interview. Eligibility included Afghan males and females at least 15 years old who were residents of the household and those who had given consent to participate in the study. The study aimed to estimate prevalence of common mental health problems in the population aged 15 or older; thus, the study was powered to estimate predictors of an outcome with prevalence of at most 20% based on existing global literature. Based on an estimated 20% outcome, minimum total sample size per region was 246 using simple random sampling assumptions; because our design was multistage cluster sampling, considering the design effect, and anticipating the non-response to be 10%, the final target sample size in each region was 542, rendering a total sample size for the country of 4475 head of family members and 4474 individuals. A consent form was read aloud and accepted for each selected person before completion; those who did not accept were excluded. Sampling weights were created based on the age and gender of the population based on census data and applied to the sample to approximate national distributions. A team of one female and one male was responsible to collect data from each household. Data collection was supervised by provincial supervisors, and regular monitoring visits were conducted by monitoring officers. Furthermore, the provincial public health directorate also conducted supervisory visits from the data collection process. Details of the study design and methodology can be found elsewhere (Kovess-Masfety et al. 2021a, b).

The distributions of study demographics are found in our previous publications (Kovess-Masfety et al. 2021a). Briefly, 52.62% of the weighted individual sample completed no formal education and did not have any reading skills, 3.5% did not complete primary school, 6.4% completed primary, 8.47% secondary, 18.1% college, and 7.85% university. 53.5% of the sample declared no income, with distinct differences by sex; 84.61% of women and 22.62% of men reported no income. 13.68% of the sample reported working in agriculture or animal husbandry, 13.8% as a laborer, 9.11% salaried, and 3.7% in business or trading. Income was very much linked to the type of employment: those in agriculture, farming, or as laborer earned an average of 7200 AFS (100$), whereas those in business earn 12,982 AFS (200$). Female disadvantage persisted for women who earned income: 42% of women who worked were in the lowest income category versus 16% of men. Urban people reported to higher-income groups compared with rural people. On the total weighted sample, 27.55% were Tajik, 47.82% were Pashtu, 11.4% Hazara, 6.58% Uzbek, 6.65% another ethnicity (for 0.08% the information was missing). As expected, ethnicities were very different across regions (Kovess-Masfety et al. 2021a).

### Instruments

The questionnaire collected pertinent socio-demographic information including gender, age, educational level, marital status, occupation (position and sector), income, and ethnicity.

Information about major depressive episodes and generalized anxiety was obtained by Composite International Diagnostic Interview Short Form (CIDI SF) (Kessler et al. 1998) to estimate DSM-5 classifications. Post-traumatic stress disorder was assessed with the life event checklist 5 (LEC-5) together with PTSD Check-List 5 (PCL) (Blevins et al. 2015; Weathers et al. 2013) using the DSM-5 algorithm.

We categorized traumatic events in five groups: collective violence, sexual violence, accidental injury, cause/witnessed harm, and interpersonal violence. First, collective violence included those who experience or witness (1) fire or explosion; (2) assault with a weapon (for example, being shot, stabbed, threatened with a knife, gun, bomb); (3) combat or exposure to a warzone (in the military or as a civilian); and (4) captivity (for example, being kidnapped, abducted, held hostage, prisoner of war). Second, sexual violence included experience or witness of (1) sexual assault (rape, attempted rape, made to perform any type of sexual act through force or threat of harm); and (2) other unwanted or uncomfortable sexual experience (for example, doing sex during ministration, doing sex without your permission with your partner). Third, accidental injury included experience or witness of (1) natural disaster (for example, flood, earthquake); (2) transportation accident (for example, car accident, plane crash); (3) serious accident at work, home, or during any activity; (4) exposure to toxic substance (for example, mercury, benzene); and (5) life-threatening illness or injury; and only witnessed of sudden accidental death. Forth, cause/witnessed harm included witness of (1) sudden violent death (for example, homicide, suicide) and (2) sudden accidental death. Fifth, interpersonal violence included experience or witness of physical assault (for example, being attacked, hit, slapped, kicked, beaten up).

Five items queried suicidal behaviors: overall death preference or if the respondent wished to die; lifetime suicidal ideation, plan, and attempt; and last 12-months suicidal ideation. Responses options included yes, no, do not know, and refused. In addition, questions were asked about suicidal thoughts and intensity of desire to die, including frequency and recency. Missing data, including responding ‘don’t know’ or refusing to answer, in these questions were relatively rare, ranging from 2 respondents missing on past 12-month suicidal ideation to 65 respondents missing on lifetime suicidal ideation. These individuals were excluded from analysis. These five measures of suicidal behavior are standard in other surveys, range in prevalence (e.g., suicidal ideation is typically common in most countries, whereas suicide attempts are more rare), and time frame (e.g., past year, lifetime) and capture distinct aspects of the suicidal process.

Information about substance abuse was collected by the Alcohol, Smoking, and Substance Involvement Screening Test (ASSIST) (World Health Organization 2010). Information on cigarette smoking, sedative use, and any other drug use was assessed, and problems related to substance use disorders were categorized based on established cutpoints for the ASSIST into: no risk, moderate, and high risk for smoking, sedative use, and any addiction.

Psychotic experiences included items that queried whether respondents saw a vision that is not really seen by other people; heard voices that other people could not hear; felt that their mind is controlled by others; felt that his/her mind was being taken by strange forces; had experience of attempts at communication from strange forces; and finally whether the respondent believed that there was a plot to harm them.

### Data management and analysis

All data were entered twice in the Census and Survey Processing System (CS-Pro), and both datasets were verified for any consistency. If inconsistencies were found, the original questionnaire was re-checked to validate the response and the corrective measures were taken. Finally, the clean dataset was integrated into STATA and further cleaning processes were conducted by a statistician and analyst.

Analyses were done with STATA 17, and all analyses incorporated sampling weights. First, frequencies and prevalences were estimated for all study variables, including outcome variables, common mental health disorders (major depression, generalized anxiety, and PTSD), any substance abuse, and traumatic events. Second, the outcome variables were cross-tabulated by independent variables to assess any bivariate relationship. Finally, logistic regression was run to determine the strength of association, both unadjusted and adjusted for demographics and co-occurring mental health and substance use.

## Results

In the total sample, 2.2% reported suicidal ideation in the past 12 months, and 18.0% reported that they currently prefer death. By sex, 1.8% (95% 1.2–2.5%) of men and 2.3% (95% CI 1.8–2.8) of women reported suicidal ideation in the past 12 months; 14.3% of men (95% CI 12.7–16.0%) and 22.4% of women (95% CI 20.7–24.2%) reported death preference. When queried across the lifetime, 7.1% of respondents reported that they had suicidal ideation at some point in their lives; 3.9% reported making a suicide plan; and 3.4% reported a suicide attempt. By sex, 5.4% (95% CI 4.4–6.6%) of men and 9.1% (95% CI 8.0–10.4) of women reported suicidal ideation in their lifetime; 4.8% of men (95% CI 2.4–4.1%) and 4.8% of women (95% CI 3.4–4.6%) reported making a suicide plan in their lifetime; and 2.3% of men (95% CI 1.7–3.1%) and 4.6% of women (95% CI 3.8–5.5%) reported making a suicide plan in their lifetime.

Table 1 shows the association between each of the suicidal behavior variables with study sample demographics. Generally, suicidal behavior was more common among women compared with men, those with Tajik and Hazara ethnicity (as compared with Pashtun, Uzbek, and other), no or low education, and poor economic status. Regional differences were apparent, with the highest levels of suicidal behavior across outcomes for those in the central and south regions, comprising the provinces of Kabul and Parwan, and Paktika and Khost, respectively.

**Table 1:** Demographic distributions of suicidal behavior in the Afghan general population in 2017

Variables	Wish for death			Lifetime suicidal ideation			Lifetime suicidal plan			Lifetime suicidal attempt			Last 12-months suicidal ideation		
	*n*	% row	*P*-value	*n*	% row	*P*-value	*n*	% row	*P*-value	*n*	% row	*P*-value	*n*	% row	*P*-value
Sex															
Male	1851	14.59	< 0.001	1848	5.25	< 0.001	1846	3.14	0.003	1847	2.33	< 0.001	1847	1.73	0.014
Female	2532	22.91		2533	9.24		2526	4.99		2530	4.58		2532	2.88	
Age															
15–24 years	1087	17.57	0.059	1087	8.19	0.053	1084	3.87	0.095	1087	3.59	0.679	1087	2.30	0.231
25–34 years	1032	17.83		1030	6.12		1029	4.28		1027	3.70		1029	2.53	
35–44 years	967	21.3		964	9.13		961	5.52		963	4.15		964	3.11	
45 + years (45–95 years)	1285	20.62		1288	6.99		1286	3.42		1288	3.18		1287	1.79	
Ethnicity															
Tajik	1176	20.83	0.004	1176	5.87	< 0.001	1174	3.32	0.001	1176	2.81	< 0.001	1176	1.87	0.01
Pashtun	2111	18.9		2105	9.69		2099	5.48		2101	4.90		2104	3.23	
Hazara	485	22.27		487	5.75		486	2.26		487	1.44		487	1.03	
Uzbek	288	11.81		287	3.14		287	2.44		287	1.74		287	1.74	
Other	282	18.44		284	5.99		284	3.17		284	3.17		283	1.41	
Education															
No education	2679	20.98	< 0.001	2672	8.35	0.001	2666	4.8	0.003	2669	4.16	0.01	2670	2.77	0.034
Less than high school	737	20.76		742	8.49		740	4.73		742	3.91		742	2.56	
High school and University	944	13.88		945	4.66		944	2.22		944	2.01		945	1.27	
Marital status															
Not married, divorced or separated	1060	19.34	0.940	1058	8.32	0.294	1056	3.98	0.653	1058	3.4	0.633	1057	2.08	0.432
Married and living with spouse	3312	19.44		3312	7.34		3305	4.30		3308	3.72		3311	2.51	
Self-reported economic status															
Very poor and poor	1691	23.71	< 0.001	1686	10.38	< 0.001	1682	5.65	< 0.001	1683	5.17	< 0.001	1686	3.68	< 0.001
Middle and rich	2627	16.48		2630	5.78		2626	3.31		2629	2.62		2628	1.6	
Region															
Central	524	32.25	< 0.001	530	10.75	< 0.001	528	6.63	< 0.001	530	5.66	< 0.001	530	3.77	< 0.001
South	552	23.37		552	14.86		550	8.18		552	8.15		551	5.26	
East	554	10.11		552	5.43		549	2.73		549	3		552	1.63	
Southwest	556	14.21		555	6.85		555	4.68		555	3.78		555	2.88	
West	544	31.07		554	10.47		552	4.89		554	3.97		554	2.53	
North	553	12.30		552	1.63		552	1.27		552	1.09		551	0.73	
Central Highland	545	22.02		534	6.55		534	3.00		533	2.44		534	0.94	
Northeast	556	10.79		553	3.98		553	2.35		553	1.45		553	1.45	
Area of residence															
Urban	1146	20.51	0.266	1147	6.97	0.388	1145	3.93	0.586	1147	3.49	0.761	1200	2.44	0.737
Rural	3238	18.99		3235	7.76		3228	4.31		3231	3.68		3180	2.27	

Table 2 shows the association between traumatic event exposures and psychiatric disorders with suicidal behavior in the Afghan general population. There were strong and consistent associations between exposure to violence and trauma, and experiences of psychiatric disorders, with suicidal behavior. For example, suicidal thoughts were most prevalence among people who experience sexual violence (prevalence of suicidal thoughts, 33.3%, *p* < 0.01 for comparison with prevalence among those who did not), those who experienced caused/witnessed harm (28.1%, *p* < 0.01), and those who witnessed sexual violence (20.9%, *p* < 0.01). Similar patterns were evident across suicidal behavior outcomes. Every psychiatric disorder also increased the risk of suicidal behavior. For example, 29.6% of those with PTSD reported suicidal ideation, compared with 6.1% of those without PTSD (*p* < 0.01). Associations were also evidence for an increase in prevalence among those with major depressive episodes, generalized anxiety disorder, psychotic experiences, smoking, and sedative addiction.

**Table 2:** Association between traumatic event exposure and psychiatric disorders in the Afghan general population in 2017

Variables	Wish for death			Lifetime suicidal ideation			Lifetime suicidal plan			Lifetime suicidal attempt			Last 12-months suicidal ideation		
	*n*	% row	*P*-value	*n*	% row	*P*-value	*n*	% row	*P*-value	*n*	% row	*P*-value	*n*	% row	*P*-value
I. Traumatic events															
Collective violence (total)															
No	1767	15.51	< 0.001	1762	5.16	< 0.001	1760	2.78	< 0.001	1762	2.16	< 0.001	1761	1.53	0.002
Yes	2597	22.14		2600	9.19		2593	5.17		2596	4.62		2599	2.96	
Collective violence (experienced)															
No	3166	17.56	< 0.001	3165	6.26	< 0.001	3160	3.48	< 0.001	3163	3.04	0.001	3164	1.99	0.007
Yes	1185	24.47		1184	11.06		1180	6.10		1182	5.16		1183	3.38	
Collective violence (witnessed)															
No	2033	16.58	< 0.001	2031	5.86	< 0.001	2028	3.11	0.001	2031	2.46	< 0.001	2029	1.68	0.004
Yes	2326	21.93		2326	9.03		2320	5.13		2322	4.61		2326	3.01	
Caused/witnessed harm (total)															
No	3744	17.44	< 0.001	3745	6.46	< 0.001	3736	3.4	< 0.001	3742	2.83	< 0.001	3743	1.87	< 0.001
Yes	614	31.43		612	14.54		612	9.31		611	8.67		612	5.72	
Caused/witnessed harm (experienced)															
No	4330	19.19	0.002	4328	7.39	< 0.001	4319	4.12	< 0.001	4325	3.58	0.05*	4326	2.36	0.07*
Yes	38	39.47		39	28.21		39	15.38		38	10.53		39	7.69	
Caused/witnessed harm (witnessed)															
No	3765	17.58	< 0.001	3766	6.59	< 0.001	3757	3.49	< 0.001	3762	2.87	< 0.001	3764	1.91	< 0.001
Yes	593	31.03		591	14.04		591	8.97		591	8.63		591	5.58	
Inter-personal violence (total)															
No	2554	16.33	< 0.001	2555	4.93	< 0.001	2550	2.08	< 0.001	2555	1.80	< 0.001	2554	1.06	< 0.001
Yes	1809	23.66		1806	11.30		1802	7.21		1802	6.22		1805	4.32	
Inter-personal violence (experienced)															
No	3292	17.13	< 0.001	3295	5.13	< 0.001	3290	2.43	< 0.001	3294	2.13	< 0.001	3294	1.34	< 0.001
Yes	1071	26.24		1066	15.10		1062	9.7		1063	8.28		1065	5.73	
Inter-personal violence (witnessed)															
No	2981	18.82	0.178	2978	7.02	0.044	2972	3.87	0.106	2978	3.29	0.082	2976	2.28	0.434
Yes	1382	20.55		1383	8.75		1380	4.93		1379	4.35		1383	2.68	
Sexual violence (total)															
No	4112	18.14	< 0.001	4111	6.76	< 0.001	4104	3.61	< 0.001	4107	3.04	< 0.001	4109	1.92	< 0.001
Yes	203	48.77		202	25.25		200	17.5		202	16.34		202	12.38	
Sexual violence (experienced)															
No	4218	18.82	< 0.001	4217	7.04	< 0.001	4210	3.87	< 0.001	4213	3.30	< 0.001	4,215	2.14	< 0.001
Yes	97	52.58		96	33.33		94	21.28		96	19.79		96	14.58	
Sexual violence (witnessed)															
No	4185	18.71	< 0.001	4183	7.22	< 0.001	4174	3.88	< 0.001	4179	3.3	< 0.001	4181	2.15	< 0.001
Yes	129	48.06		129	20.93		129	16.28		194	15.5		129	10.85	
Accidental injury (total)															
No	958	11.8	< 0.001	958	4.8	< 0.001	958	2.40	0.001	958	1.88	0.001	958	1.77	0.148
Yes	3409	21.56		3407	8.34		3398	4.74		3403	4.14		3405	2.58	
Accidental injury (experienced)															
No	2183	14.38	< 0.001	2181	5.50	< 0.001	2181	3.07	< 0.001	2181	2.48	< 0.001	2181	1.7	0.002
Yes	2165	24.43		2165	9.70		2156	5.43		2161	4.86		2163	3.14	
Accidental injury (witnessed)															
No	1401	15.77	< 0.001	1407	6.25	0.025	1406	3.41	0.071	1407	3.13	0.202	1406	2.2	0.540
Yes	2958	21.06		2950	8.17		2942	4.59		2946	3.9		2949	2.51	
II. Common mental health disorders															
Depression (f32cor)															
No	3780	14.44	< 0.001	3779	4.9	< 0.001	3774	2.49	< 0.001	3775	2.04	< 0.001	3778	1.14	< 0.001
Yes	537	52.14		536	26.31		533	16.7		536	14.93		535	11.4	
General anxiety disorder (f41cor)															
No	4213	17.87	< 0.001	4212	6.43	< 0.001	4203	3.43	< 0.001	4208	2.95	< 0.001	4210	1.83	< 0.001
Yes	159	56.6		158	36.08		158	24.05		158	20.89		158	17.09	
PTSD															
No	4122	17.30	< 0.001	4118	6.14	< 0.001	4113	3.33	< 0.001	4114	2.80	< 0.001	4116	1.70	< 0.001
Yes	262	52.29		264	29.55		260	18.08		264	16.67		264	13.26	
Lifetime psychotic idea															
No	2,868	14.33	< 0.001	2876	4.31	< 0.001	2872	1.88	< 0.001	2873	1.43	< 0.001	2876	0.90	< 0.001
Yes	1111	32.31		1112	14.75		1109	9.65		1111	8.64		1110	6.31	
Smoking risk															
No or low risk	3449	18.7	< 0.001	3447	6.9	0.002	3441	3.81	< 0.001	3445	3.48	0.001	3446	2.15	< 0.001
Moderate risk	827	20.31		828	9.42		825	4.85		826	3.39		827	2.42	
High risk	107	34.58		106	14.15		106	12.26		106	10.38		106	10.38	
Sedative use															
No or low risk	4048	18.08	< 0.001	4043	6.51	< 0.001	4036	3.59	< 0.001	4040	3.09	< 0.001	4042	2.00	< 0.001
Moderate risk	262	31.30		265	15.09		263	7.98		264	6.44		265	4.15	
High risk	70	50.00		70	40.00		70	25.71		70	24.29		69	18.84	
Any addiction															
No or low risk	3189	17.53	< 0.001	3186	6.12	< 0.001	3181	3.33	< 0.001	3185	3.01	< 0.001	3186	1.88	< 0.001
Moderate risk	1001	21.68		1004	9.06		1000	4.60		1001	3.4		1003	2.09	
High risk	194	38.14		192	23.44		192	16.67		192	15.1		191	12.57	

*Fishers exact test

Table 3 describes the association between demographic characteristics and suicidal behavior outcomes in logistic regression models with all demographic characteristics in the model. Overall, women were more likely to report suicidal behavior than men, with the strongest association for lifetime suicide attempt (OR = 1.83; 95% CI 1.20–2.79). Suicidal behavior did not vary substantially by age, although those 45 + years were more likely to wish for death than those in the youngest age group (OR = 1.40; 95% CI 1.06–1.83). Few associations emerged by ethnicity, although compared with Tajik ethnicity, those reporting Pashtun ethnicity were more likely to report lifetime suicidal thoughts (OR = 1.74; 95% CI 1.14–2.66). Higher levels of education were associated with lower odds of suicidal behavior, with the strongest association for lifetime suicidal planning (OR = 0.51; 95% CI 0.27–0.94). Economic status was robustly associated with suicidal behavior; for example, those reporting that they are middle class and rich have 0.43 times the odds of past 12-month suicidal ideation compared with those who report that they are poor or very poor (95% CI 0.27–0.67). Few associations emerged for urban versus rural residence or by marital status.

**Table 3:** Adjusted association between demographic characteristics with suicidal behavior in the Afghan general population in 2017

Variables	Wish for death		Lifetime suicidal ideation		Lifetime suicidal plan		Lifetime suicidal attempt		Last 12-months suicidal ideation	
	OR (95% CI)	*P*-value	OR (95% CI)	*P*-value	OR (95% CI)	*P*-value	OR (95% CI)	*P*-value	OR (95% CI)	*P*-value
Sex (Ref = Male)										
Female	1.574 [1.302, 1.902]	0.000	1.581 [1.18, 2.118]	0.002	1.263 [0.858, 1.86]	0.236	1.829 [1.197, 2.794]	0.005	1.271 [0.752, 2.148]	0.37
Age (Ref = 15–24 years)										
25–34 years	1.044 [0.789, 1.382]	0.760	0.725 [0.485, 1.085]	0.118	1.046 [0.62, 1.764]	0.866	0.857 [0.487, 1.508]	0.593	0.992 [0.545, 1.806]	0.979
35–44 years	1.302 [0.973, 1.741]	0.075	0.911 [0.609, 1.361]	0.647	1.101 [0.645, 1.88]	0.723	0.829 [0.466, 1.477]	0.526	0.944 [0.515, 1.73]	0.853
45 + yrs (45–95 Years)	1.396 [1.063, 1.834]	0.016	0.725 [0.495, 1.06]	0.097	0.696 [0.421, 1.151]	0.158	0.646 [0.384, 1.085]	0.099	0.567 [0.312, 1.032]	0.063
Ethnicity (Ref = Tajik)										
Pashtun	1.067 [0.812, 1.403]	0.640	1.74 [1.139, 2.658]	0.010	1.485 [0.863, 2.556]	0.154	1.288 [0.712, 2.33]	0.402	1.416 [0.651, 3.083]	0.380
Hazara	1.053 [0.737, 1.504]	0.777	1.066 [0.567, 2.003]	0.843	0.719 [0.291, 1.775]	0.474	0.418 [0.15, 1.165]	0.095	0.882 [0.267, 2.914]	0.836
Uzbek	1.106 [0.695, 1.758]	0.671	0.86 [0.338, 2.186]	0.751	1.339 [0.445, 4.035]	0.603	1.47 [0.387, 5.581]	0.571	1.312 [0.398, 4.327]	0.656
Other	1.224 [0.823, 1.821]	0.318	2.079 [1.101, 3.927]	0.024	2.016 [0.873, 4.655]	0.101	2.123 [0.928, 4.858]	0.075	1.217 [0.335, 4.425]	0.765
Education (Ref = No education)										
Less than high school	1.006 [0.784, 1.291]	0.963	1.047 [0.724, 1.514]	0.806	1.085 [0.681, 1.73]	0.730	1.062 [0.64, 1.764]	0.815	1.067 [0.575, 1.98]	0.837
High school and university	0.735 [0.559, 0.965]	0.027	0.641 [0.419, 0.981]	0.040	0.506 [0.273, 0.938]	0.030	0.614 [0.328, 1.152]	0.129	0.475 [0.203, 1.116]	0.088
Marital status (Ref = Not married and widowed or separated)										
Married and living with spouse	0.945 [0.737, 1.213]	0.658	0.862 [0.608, 1.221]	0.404	0.944 [0.578, 1.542]	0.818	1.054 [0.62, 1.793]	0.845	0.905 [0.516, 1.586]	0.727
Self-reported economic status (Ref = Very poor and poor)										
Middle and rich	0.61 [0.513, 0.727]	0.000	0.514 [0.397, 0.665]	0.000	0.573 [0.41, 0.8]	0.001	0.49 [0.342, 0.702]	0.000	0.428 [0.273, 0.671]	0.000
Region (Ref = Central)										
South	0.453 [0.315, 0.653]	0.000	0.855 [0.505, 1.446]	0.558	0.781 [0.414, 1.47]	0.443	1.111 [0.557, 2.215]	0.766	0.882 [0.371, 2.097]	0.776
East	0.209 [0.138, 0.317]	0.000	0.273 [0.149, 0.5]	0.000	0.242 [0.11, 0.532]	0.000	0.31 [0.134, 0.717]	0.006	0.298 [0.104, 0.851]	0.024
Southwest	0.235 [0.162, 0.34]	0.000	0.357 [0.208, 0.611]	0.000	0.355 [0.195, 0.649]	0.001	0.396 [0.202, 0.776]	0.007	0.406 [0.18, 0.915]	0.030
West	0.708 [0.52, 0.964]	0.028	0.643 [0.397, 1.042]	0.073	0.489 [0.264, 0.903]	0.022	0.506 [0.258, 0.995]	0.048	0.423 [0.186, 0.959]	0.039
North	0.255 [0.173, 0.376]	0.000	0.11 [0.05, 0.245]	0.000	0.146 [0.058, 0.368]	0.000	0.136 [0.052, 0.356]	0.000	0.158 [0.046, 0.544]	0.003
Central highland	0.479 [0.329, 0.697]	0.000	0.498 [0.265, 0.935]	0.030	0.463 [0.204, 1.051]	0.066	0.586 [0.244, 1.407]	0.232	0.195 [0.055, 0.687]	0.011
Northeast	0.214 [0.138, 0.33]	0.000	0.342 [0.162, 0.723]	0.005	0.275 [0.102, 0.737]	0.010	0.224 [0.067, 0.744]	0.015	0.297 [0.092, 0.96]	0.043
Area of residence (Ref = Urban)										
Rural	1.123 [0.879, 1.435]	0.353	1.027 [0.685, 1.54]	0.896	1.068 [0.628, 1.816]	0.808	0.981 [0.554, 1.738]	0.948	1.005 [0.484, 2.087]	0.989

Adjusted for sex, age, ethnicity, education, marital status, self-reported economic status, region, and urbanicity

In Table 4, we report the association between traumatic event exposures and suicidal behavior, adjusted for sex, age, ethnicity, education, marital status, economic status (self-reported), regions and residential area (urban and rural). There were strong and statistically significant associations among almost all independent variables with the range of suicidal behavior outcomes. The strongest magnitude of associations was observed for experiences of sexual violence. For example, respondents who reported experiencing sexual violence were 4.1 times more likely to report death preference (95% CI 2.56–6.44), 3.7 times more likely to report lifetime suicidal ideation (95% CI 2.2–6.2), 4.2 times more likely to report lifetime suicide plan (95% CI 2.2–7.9), 4.4 times more likely to report lifetime suicide attempt (95% CI 2.3–8.4), and 5.8 times more likely to report past 12-month suicidal ideation (95% CI 2.7–12.4) compared with those who did not report experiencing sexual violence. Other traumatic event exposures also increased risk of suicidal behavior, with odds ratios generally ranging from approximately 1.2 to 3–4 times increased risk. Associations for witnessing trauma, such as accidental injury and collective violence, generally were lower in magnitude than associations for experiencing trauma, although even witnessing trauma was associated with increased risk.

**Table 4:** Adjusted association between traumatic event exposure with suicidal behavior in the Afghan general population in 2017

Variables	Wish for death		Lifetime suicidal ideation		Lifetime suicidal plan		Lifetime suicidal attempt		Last 12-months suicidal ideation	
	OR (95% CI)	*P*-value	OR (95% CI)	*P*-value	OR (95% CI)	*P*-value	OR (95% CI)	*P*-value	OR (95% CI)	*P*-value
Collective violence (total) (Ref = No)										
Yes	1.792 [1.476, 2.175]	0.000	2.004 [1.491, 2.694]	0.000	2.321 [1.579, 3.411]	0.000	2.866 [1.877, 4.377]	0.000	2.05 [1.245, 3.374]	0.005
Collective violence (experienced) (Ref = No)										
Yes	1.732 [1.424, 2.107]	0.000	2.467 [1.844, 3.3]	0.000	2.434 [1.67, 3.548]	0.000	2.739 [1.845, 4.065]	0.000	2.517 [1.547, 4.097]	0.000
Collective violence (witnessed) (Ref = No)										
Yes	1.639 [1.363, 1.972]	0.000	1.725 [1.311, 2.268]	0.000	2.08 [1.455, 2.974]	0.000	2.565 [1.73, 3.804]	0.000	2.1 [1.306, 3.376]	0.002
Caused/witnessed harm (total) (Ref = No)										
Yes	2.322 [1.842, 2.928]	0.000	2.09 [1.543, 2.831]	0.000	2.336 [1.605, 3.4]	0.000	2.719 [1.835, 4.031]	0.000	2.578 [1.605, 4.142]	0.000
Caused/witnessed harm (experienced) (Ref = No)										
Yes	2.87 [1.429, 5.76]	0.003	4.784 [2.154, 10.627]	0.000	3.811 [1.364, 10.648]	0.011	3.777 [1.06, 13.452]	0.040	2.375 [0.434, 12.993]	0.318
Caused/witnessed harm (witnessed) (Ref = No)										
Yes	2.216 [1.751, 2.805]	0.000	1.905 [1.398, 2.596]	0.000	2.076 [1.422, 3.029]	0.000	2.543 [1.715, 3.77]	0.000	2.369 [1.463, 3.834]	0.000
Inter-personal violence (total) (Ref = No)										
Yes	1.779 [1.484, 2.134]	0.000	2.332 [1.759, 3.09]	0.000	3.569 [2.426, 5.25]	0.000	3.784 [2.491, 5.75]	0.000	4.185 [2.495, 7.022]	0.000
Inter-personal violence (experienced) (Ref = No)										
Yes	1.731 [1.434, 2.091]	0.000	2.842 [2.158, 3.741]	0.000	3.889 [2.724, 5.553]	0.000	3.897 [2.636, 5.762]	0.000	3.992 [2.488, 6.406]	0.000
Inter-personal violence (witnessed) (Ref = No)										
Yes	1.309 [1.079, 1.589]	0.006	1.197 [0.904, 1.585]	0.209	1.306 [0.907, 1.88]	0.152	1.46 [0.981, 2.174]	0.062	1.203 [0.737, 1.962]	0.460
Sexual violence (total) (Ref = No)										
Yes	4.52 [3.168, 6.448]	0.000	2.837 [1.889, 4.261]	0.000	3.957 [2.425, 6.456]	0.000	4.217 [2.574, 6.909]	0.000	5.625 [3.148, 10.052]	0.000
Sexual violence (experienced) (Ref = No)										
Yes	4.065 [2.564, 6.444]	0.000	3.647 [2.158, 6.163]	0.000	4.189 [2.21, 7.942]	0.000	4.402 [2.307, 8.397]	0.000	5.777 [2.698, 12.37]	0.000
Sexual violence (witnessed) (Ref = No)										
Yes	4.66 [3.004, 7.228]	0.000	2.456 [1.464, 4.12]	0.001	3.909 [2.191, 6.976]	0.000	4.263 [2.352, 7.727]	0.000	4.835 [2.37, 9.865]	0.000
Accidental injury (total) (Ref = No)										
Yes	2.423 [1.906, 3.081]	0.000	1.977 [1.374, 2.845]	0.000	2.431 [1.466, 4.033]	0.001	3.479 [2.039, 5.935]	0.000	1.618 [0.888, 2.947]	0.116
Accidental injury (experienced) (Ref = No)										
Yes	1.881 [1.555, 2.275]	0.000	1.966 [1.495, 2.587]	0.000	1.852 [1.277, 2.686]	0.001	2.285 [1.517, 3.443]	0.000	1.902 [1.151, 3.144]	0.012
Accidental injury (witnessed) (Ref = No)										
Yes	1.72 [1.408, 2.1]	0.000	1.43 [1.06, 1.929]	0.019	1.399 [0.936, 2.093]	0.102	1.476 [0.962, 2.264]	0.075	1.128 [0.672, 1.894]	0.648

Adjusted for sex, age, ethnicity, education, marital status, self-reported economic status, region, and urbanicity

Table 5 shows the associations between psychiatric disorders and suicide risk adjusted for sex, age, ethnicity, education, marital status, economic status (self-reported), regions and residential area (urban and rural), and traumatic event categories. Associations were strong and significant for all psychiatric disorders related to suicidal behavior risk. The strongest associations were observed for past 12-month suicidal ideation. Respondents who met criteria for major depressive episodes (OR = 7.48; 95% CI 4.40–12.72), generalized anxiety disorder (OR = 6.61; 95% CI 3.54–12.33), PTSD (OR = 7.26; 95% CI 4.21–12.51), psychotic experiences (OR = 5.64; 95% CI 3.22–9.87), high smoking addiction risk (OR = 4.39; 95% CI 1.87–10.32), and high sedative addiction risk (OR = 5.60; 95% CI 2.64–11.88) had the highest risk of past 12-month suicidal ideation. Across all disorders and suicidal behavior outcomes, experiencing psychiatric disorders increased the risk of suicidal behavior, with odds ratios ranging from approximately 1.2 to 7–8 times increases in risk. Sex remained a significant risk factor in all models, with women at higher risk than men. Odds ratios for the increase in risk for women in these adjusted analyses ranged from (OR = 1.27; 95% CI 0.75, 2.15) for last 12 months suicidal ideation to (OR = 1.58; 95% CI 1.18, 2.12) for lifetime suicidal ideation.

**Table 5:** Adjusted association between psychiatric disorders with suicidal behavior in the Afghan general population in 2017

Variables	Wish for death		Lifetime suicidal ideation		Lifetime suicidal plan		Lifetime suicidal attempt		Last 12-months suicidal ideation	
	OR (95% CI)	*P*-value	OR (95% CI)	*P*-value	OR (95% CI)	*P*-value	OR (95% CI)	*P*-value	OR (95% CI)	*P*-value
Depression (Ref = No)										
Yes	5.42 [4.25, 6.91]	0.000	5.36 [3.95, 7.29]	0.000	5.29 [3.53, 7.94]	0.000	5.59 [3.64, 8.60]	0.000	7.48 [4.40, 12.72]	0.000
General anxiety disorder (Ref = No)										
Yes	3.89 [2.62, 5.77]	0.000	5.38 [3.56, 8.11]	0.000	5.48 [3.38, 8.90]	0.000	5.18 [3.08, 8.70]	0.000	6.61 [3.54, 12.33]	0.000
PTSD (Ref = No)										
Yes	4.05 [2.96, 5.54]	0.000	5.02 [3.50, 7.22]	0.000	4.51 [2.86, 7.11]	0.000	4.79 [2.95, 7.79]	0.000	7.26 [4.21, 12.51]	0.000
Lifetime psychotic experiences (Ref = No)										
Yes	2.15 [1.76, 2.63]	0.000	2.84 [2.08, 3.88]	0.000	4.04 [2.65, 6.14]	0.000	4.86 [3.07, 7.70]	0.000	5.64 [3.22, 9.87]	0.000
Smoking risk (Ref = No or low risk)										
Moderate risk	1.39 [1.09, 1.77]	0.008	1.87 [1.30, 2.69]	0.001	1.57 [0.99, 2.48]	0.053	1.27 [0.76, 2.13]	0.365	1.41 [0.73, 2.73]	0.308
High risk	2.32 [1.40, 3.84]	0.001	2.04 [1.00, 4.16]	0.050	2.92 [1.39, 6.13]	0.005	2.54 [1.06, 6.10]	0.037	4.39 [1.87, 10.32]	0.001
Sedative use (Ref = No or low risk)										
Moderate risk	1.72 [1.24, 2.38]	0.001	2.50 [1.61, 3.86]	0.000	2.14 [1.25, 3.64]	0.005	2.12 [1.12, 4.01]	0.020	1.69 [0.82, 3.45]	0.153
High risk	2.69 [1.55, 4.68]	0.000	6.11 [3.44, 10.87]	0.000	4.91 [2.55, 9.45]	0.000	4.88 [2.50, 9.51]	0.000	5.60 [2.64, 11.88]	0.000
Any addiction (Ref = No or low risk)										
Moderate risk	1.50 [1.20, 1.86]	0.000	1.87 [1.35, 2.60]	0.000	1.60 [1.04, 2.45]	0.032	1.41 [0.88, 2.28]	0.155	1.24 [0.66, 2.35]	0.503
High risk	2.42 [1.67, 3.50]	0.000	3.85 [2.43, 6.09]	0.000	4.13 [2.41, 7.07]	0.000	3.92 [2.18, 7.06]	0.000	5.44 [2.80, 10.55]	0.000

Adjusted for sex, age, ethnicity, education, marital status, self-reported economic status, regions and residential area (urban and rural), collective violence (total), caused/witnessed harm (total), interpersonal violence (total), sexual violence (total), accidental injury (total)

## Discussion

The present study documents the high burden of suicidal behavior in the general population of Afghanistan, and the association with established risk factors for suicide including psychiatric disorders and experiences of trauma. We document strong and significant associations with traumatic event exposures, especially those involving sexual violence, with suicidal behavior, as well as associations with psychiatric disorders across a broad range of symptoms, including depressive, anxiety, psychotic, and substance use disorders. To date, this is the largest psychiatric epidemiological study of the Afghan general population conducted, and we document the need for increased mental health support infrastructure throughout the country. Given that political events have escalated in the country, especially in 2021, a continued focus on Afghan mental health is critical to public health in the regions. Suicide, including both death by suicide and injuries that result from intentional self-harm, continues to be major drivers of morbidity and mortality worldwide, and outcomes that are preventable with accessible mental health and social support. Ensuring that such supports are in place across the world, including vulnerable regions such as Afghanistan, is critical to building global mental health.

The findings from the general population of Afghanistan are higher than with those from other low- to middle-income regions; Borges et al. document 12-month ideation rates of 2.4% (SE = 0.1) for females and 1.6% (SE = 0.1) for males (Borges et al. 2010). Rates for low- and middle-income countries are generally and slightly lower than for high-income countries, which have prevalence rates of 2.2% for females and 1.7% for males for 12-month suicidal ideation. A European multi-country study showed large differences across European countries, especially among women, with rates consistent with the rate of suicidal ideation among Afghan women (e.g., 14.9% in France, 13.1% in Portugal) as well as attempts (e.g., 4.9% Portugal, 5.4% in France). Data on lifetime suicide attempts from studies with similar population-based methodology are less available, suggesting an important area for future research.

Reporting on data from 21 countries that were part of the WHO World Mental Health surveys, Nock et al. (2009) documented increases in the risk of suicidal behavior among those with psychiatric disorders of approximately 1.5 to threefold (Nock et al. 2009). In Afghanistan, we found many associations in a similar direction and magnitude, and even higher for proximal suicidal behavior such as past 12-month ideation. Nock et al. (2009) also found that overall, mental disorders were equally predictive in developed and developing countries, with a key difference being that the strongest predictors of suicide attempts in developed countries were mood disorders, whereas in developing countries impulse control, substance use, and post-traumatic stress disorders were most predictive (Nock et al. 2009). Results revealed that approximately 80% of suicide attempters in the USA have a temporally prior mental disorder. Anxiety, mood, impulse control, and substance use disorders all significantly predict subsequent suicide attempts; however, these associations decrease substantially in multivariate analyses controlling for comorbidity but remain statistically significant in most cases (Nock et al. 2010). Further, these results are in line with other psychiatric epidemiological studies that have documented the serious public health risk of exposure to trauma for suicidal behavior. A meta-analysis in 2018 concluded that depressive disorders are one of the strongest predicators of suicide, and increase risk for suicide ideation, attempt, and death (Ribeiro et al. 2018). Another study found that anxiety is a statistically significant, yet weak, predictor of suicide ideation and attempts, and that PTSD is among the strongest associations for suicidal behaviors (Bentley et al. 2016). Indeed, interventions to address future suicide risk among those exposed to trauma are a key area for implementation science and methodological innovation in program and treatment development. Substance use disorders are a consistent risk factor for suicidal behavior, consistent with our results (Artenie et al. 2015a, b). Our results underscore the importance of such efforts, especially in areas with high levels of trauma exposure. Studies in Afghanistan, including our own, indicate that there are mental health treatment gaps, much like there are in many areas of the world, highlighting the need for a sustained focus on mental health to be included in humanitarian response in the region.

Our results on demographic predictors of suicide are also consistent with other research, documenting that social identity and socio-economic status remain robust predictors of suicide risk throughout the world. Risk factors for suicidal behaviors in both developed and developing countries that are confirmed in these data include female sex and lower education and income (Borges et al. 2010; Qin et al. 2003). There is abundant evidence indicating that low socioeconomic position, irrespective of the economic status of the country in question, is associated with an increased risk of suicide, including the suggestion that the recent global economic recession has been responsible for an increase in suicide deaths and, by proxy, attempts. These data also indicate regional variation in suicidal behavior within Afghanistan, with the central and southern regions at particularly increased risk. Reasons for regional variation remain speculative. The central region of Afghanistan includes major urban areas such as Kabul; residents may be more willing to disclose suicidal behavior in these areas and more likely to have connected with mental health services. The southern regions have significant areas of ongoing political conflict, a high rate of immigrants, as well as burdensome living and marital conditions that place individuals at increased risk. While we can only speculate about reasons for differences across provinces, higher rates in the central region may be attributable to domestic terrorism which has targeted the region and increased fear and despair, or compositional factors such as high levels of in-migration from other provinces due to scarcity in home regions. Given that spatial clustering of suicides and suicidal behavior remains an important area of research (Keyes et al. 2021), greater emphasis on understanding spatial aspects of suicide risk in high-conflict settings such as Afghanistan is important for future research.

The mechanisms that underlie the associations between trauma exposure, psychiatric disorder, and suicide risk are well documented. Individuals enduring trauma, especially when there is grave threat to physical integrity and bodily harm, are at increased risk for feelings of hopelessness, intrusive reexperiences of trauma, and feelings of disassociation and inability to feel closeness to loved ones (Bath 2008; Ásgeirsdóttir et al. 2018; Beautrais 2002; Nolen-Hoeksema 2012). Social isolation and fear of reporting or continued harm can also render difficult emotional pain leading to suicidal crises (Nock et al. 2010; Möller-Leimkühler 2002). Further, witnessing trauma can also lead to intrusive and unpleasant thoughts and emotions, bereavement for those lost in the circumstance of violent trauma to others, a fear of retaliation, or ongoing physical threat (Panagioti et al. 2015). The magnitude of the emotional response to trauma is evident in the results that we present here, with almost all traumas increasing the risk of suicidal behavior across the lifecourse.

Suicidal behaviors were more prevalent on women than men in univariate as well as in multivariable analyses: wishing for death, lifetime suicidal thoughts, and moreover suicidal attempts occurred at approximately twice the rate in women compared to men, which is consistent with other data among Middle Eastern Muslim women (Rezaeian 2010). Higher rates of suicidal behaviors have consistently been identified among women compared with men in many other countries as well (Boyd et al. 2015).

The population have Afghanistan has endured decades and generations of political conflict that has resulted in major disruptions in economic, educational, and social institutions, and exposed the population to high levels of poverty, unemployment, and injuries due to ongoing cycles of violence. Rates of psychiatric disorders including PTSD have remained high through, and because of, these conflicts (Miller et al. 2008; Alemi et al. 2018). The effects of this ongoing, chronic exposure to stress were amplified in 2021 with another major upheaval given the change in the national political leadership to the Taliban. This political change prompted significant migration and displacement has families left the country, and for those who remained, there have been increases in financial trauma, unemployment, and food insecurity at the population level (United Nations Development Program 2021). While data on current mental health status in Afghanistan remain scarce, available data and journalism sources suggest that there have been increases in mental health distress, suicide attempts, and fatal suicide, especially among young girls, who are excluded from educational opportunities.

The political conflicts and resulting economic instability have also resulted in unmet need for treatment of psychiatric disorders and distress in mental health care systems. While again data are scarce, reports from experts in the country indicate that mental health services are increasingly underfunded after the political conflicts of 2021 and the loss of international aid sources, especially in rural areas (HealthNet 2021; Diwakar 2021). Thus, while the population of Afghanistan is increasingly burdened with psychiatric disorders and suicidal behavior, the systems to treat the population for mental health problems is increasingly compromised. Ensuring that discussions of international relief include provisions for mental health treatment are critical moving forward in the country, and having data surveillance and systems in place to track population mental health burden is critical.

Limitations of the present study should be considered. Data collection was cross-sectional; other literature has reported longitudinal results on trauma experiences, psychiatric disorders, and suicidal behavior, however none to our knowledge in the country of Afghanistan. Ongoing surveillance of mental health disorders and suicidal behavior in Afghanistan is challenging in the current political climate. Experiences were based on self-report, as is common in psychiatric epidemiological studies; however, evidence indicates that self-report of suicidal behavior is relatively reliable. Finally, the survey did not include information on all possible risk factors for suicidal behavior, including genetic vulnerability and family history. Indeed, the development of suicide risk is complex, involving contributions from biological (including genetics), psychological (such as certain personality traits), clinical (such as comorbid psychiatric illness), social and environmental factors (Turecki et al. 2019). The complexity of suicidal behavior is supported by many other studies that document how suicide is influenced by the interaction of a variety of biological, clinical, psychological, social, cultural, and environmental factors. As we conduct more research in the region, we plan to attend to these aspects of psychiatric disorder risk as well.

## Conclusions

In conclusion, the population of Afghanistan has endured substantially traumatic event exposures, with significant implications for the burden and distribution of mental health problems and suicidal behavior in the country. Continued research focused on understanding the unique experiences of people in Afghanistan, especially those with psychiatric disorders, is a critical for informing public health assessments of needs.

## Data Availability

Data are not available for public use.

## Abbreviations

ASSIST: Alcohol, Smoking, and Substance Involvement Screening Test
CIDI SF: Composite International Diagnostic Interview Short Form
CS Pro: Census and Survey Processing System
LEC 5: Life Event Checklist 5
PCL: PTSD Check-List 5
PTSD: Post-Traumatic Stress Disorder
